# Sexual Dimorphism in Abdominal Aortic Aneurysm—Insights from Clinical and Experimental Studies

**DOI:** 10.3390/jvd4010005

**Published:** 2025-01-31

**Authors:** Zain Husain Islam, Hongzhang Mei, Zoe Tetz, Rohan Kanchetty, Sophia Stanisic, Nicholas Hoyt, William Aaron Marcum, Campbell Johnston, Eric William Kent, Mengxue Zhang, Nina Islam, Alvin Anand, Kaijie Zhang, Li Yin, Bowen Wang

**Affiliations:** 1 Department of Surgery, School of Medicine, University of Virginia, Charlottesville, VA 22908, USA; 2 Department of Surgery, Feinberg School of Medicine, Northwestern University, Chicago, IL 60603, USA; 3 Department of Pathology, The University of Chicago Medicine, Chicago, IL 60637, USA; 4 Department of Vascular Surgery, Second Affiliated Hospital of Zhejiang University School of Medicine, Hangzhou 310058, China

**Keywords:** abdominal aortic aneurysm, sexual dimorphism, peripheral vascular disease

## Abstract

Abdominal aortic aneurysm (AAA) is a prevalent vascular disease with high mortality rates upon rupture. AAA features a distinct sexual dimorphism, with a prevalence three times higher in males than in females. Interestingly, females are faced with a greater risk of rupture and a worse prognosis following surgical repairs. Nevertheless, stratified approaches for managing and predicting outcomes of AAA in male and female patients remain limited, largely hindered by our incomplete understanding of the mechanisms underlying this sex dimorphism. In this article, we will summarize the recent clinical and preclinical efforts aimed at understanding the therapeutic and mechanistic implications of sex-specific factors shaping AAA.

## 1. Introduction

An abdominal aortic aneurysm (AAA) is a localized, progressive weakening and dilation of the aortic wall, primarily in the infrarenal segment [1]. AAA presents a significant cardiovascular risk and public health burden, particularly among individuals aged 65 years and older [2]. A ruptured AAA can be extremely lethal, associated with 80–90% mortality [3]. Due to increased efforts in the expanded ultrasound screening for at-risk populations, more patients with asymptomatic AAA have been diagnosed, leading to a gradual decline in AAA-related mortalities (from ~15,000 in the 2000s to <10,000 in recent years) [4]. Nevertheless, the costs of AAA and its associated complications on public health remain profound, estimated to account for a crude rate of 1 death per 100,000 in the United States. A growing body of evidence accumulated over the past decades has revealed a distinct sexual dimorphism in AAA, as summarized in Supplementary Table S1. This introduces a critical dimension to our understanding of AAA, demanding an exploration into the nuanced interplay of sex-specific factors shaping the course of this vasculopathy.

The male sex emerges as a pivotal nonmodifiable risk factor for AAA, carrying a four-fold to five-fold higher incidence compared to females and an alarming ten-fold higher risk for AAA development in males compared with age-matched females [5]. This sex discrepancy accentuates the urgency in the clinical landscape, especially considering the current absence of proven medical therapies to attenuate AAA expansion and rupture. Surgical repairs, reserved primarily for AAAs reaching a critical size of 5.5 cm, remain the cornerstone of intervention [6]. However, this highlights a critical gap in therapeutic options, particularly for smaller AAAs where a watchful waiting strategy is employed, leaving patients vulnerable to unpredictable and potentially lethal ruptures. Unfortunately, recent trials of pharmacotherapies, such as doxycycline, have all failed, testifying to the unmet clinical needs of these patients.

Intriguingly, despite being associated with a lower incidence, females face a worse prognosis upon AAA development and rupture [7]. Reports indicate faster progression and rupture at smaller sizes, culminating in elevated post-repair complications and mortality rates compared to males [5,7]. This divergence in clinical outcomes underscores the necessity for a nuanced understanding of AAA dynamics in different biological sexes, extending beyond mere prevalence statistics to encompass the entire disease trajectory.

In this review, our objective is to summarize the recent evidence—both clinical and experimental—concerning the understanding of the sexual dimorphism present in AAA. Herein, we conducted a comprehensive review of relevant literature on this topic using PubMed with the following keywords (“abdominal aortic aneurysm”, “female”, “sex differences”, “sex dimorphism”, “gender”, “experimental”, and “animal model”) published between 1990 and 2024. We limited our review to peer-reviewed articles in English (including original research articles and systemic reviews). Non-peer-reviewed articles and those published in languages other than English were excluded. We further manually reviewed the references and excluded those lacking data or discussion related to females. This exploration extends beyond prevalence statistics, incorporating clinical outcomes, epidemiological evidence, and experimental findings. Through this comprehensive approach, we strive to provide an overview of the contemporary understanding of sex-specific factors influencing the pathogenesis, diagnosis, and management of AAA, laying the foundation for future targeted interventions and personalized management strategies in this challenging vascular condition.

## 2. Clinical Evidence Concerning Sexual Dimorphism in AAA

A plethora of evidence support the idea that AAA is more likely to occur in males than in females (Table S1), with varying degrees of the “male disadvantages” throughout different studies [8]. Despite this seemingly “female advantage”, female patients experience faster AAA expansion (3.67 mm/year) in comparison to males (2.03 mm/year) as documented in The United Kingdom Small Aneurysm Trial and others [9]. Women also have a greater risk of rupture at smaller AAA diameters, and are at a higher risk of death upon rupture [10]. Moreover, females suffer from higher operative mortalities and other related harms, thereby presenting another challenge for both intact and ruptured AAA repairs in women [11]. Additionally, females also seem to develop AAA at an older age and with a more complex anatomy [10]. However, there is not one exact cause for the sexual dimorphism in the prevalence and prognosis of AAA. Differences in the actions of sex hormones, aortic anatomy, the immune system, lifestyle, and many others may critically underlie the observed difference between male and female [12]. Due to the scarcity of published reports available, below, we will primarily focus on the contemporary guidelines for sex-specific AAA diagnosis and management, as well as clinical evidence concerning the role of sex hormones, anatomical factors, and postoperative outcomes associated with the sex dimorphism in AAA.

### 2.1. Current Guidelines for Sex-Specific AAA Diagnosis and Management

Despite the significant differences in the AAA risk and prognosis between males and females, sex-stratified guidelines for AAA diagnosis and management remain scarce.

The recommendations from the Society for Vascular Surgery (SVS) and the US Preventive Services Task Force (USPSTF) both underscore the importance of sex-specific screening protocols [11,13,14]. Notably, ultrasound screening is recommended for males aged 65 to 75 with a history of smoking in both guidelines, establishing a targeted approach for a population with a higher vulnerability [5]. However, when it comes to females and the elderly (aged 75 and older), screenings are only recommended by the SVS guidelines. Strikingly, a parallel recommendation for females is absent in the 2019 USPSTF recommendation, revealing a crucial nuance in screening strategies that underscores the necessity for tailored approaches based on sex differences [11]. No specific mentioning of sex-stratified screening has been included in recent guideline updates from the European Society of Cardiology (ESC) and European Society for Vascular Surgery (ESVS) [15,16]. Notably, in response to newly identified evidence highlighting the significant risk for female smokers, the American Heart Association (AHA) and the American College of Cardiology (ACC) have expressed support for ultrasound screening for females aged 65 years and older with a history of smoking in their 2022 Joint Guidelines for the Diagnosis and Management of Aortic Disease [17,18]. These updates to screening guidelines reflect the growing recognition of women as a vulnerable population at risk for undiagnosed AAA and its potentially lethal rupture.

### 2.2. Implication of Sex Hormones

Female hormones, such as estrogen, emerge as a putative protective factor against AAA development in females [19]. This is supported by the fact that women are, on average, diagnosed and experience AAA rupture at older ages than men, with two-thirds of AAA-associated mortalities reported in females at >80 years of age [11]. This is likely due to the estrogen decrease accompanied later in life as a result of menopause in females [19,20]. Evidence also suggests that estradiol may be associated with a protective effect in women, as larger AAAs in women are linked to an earlier onset of menopause [19]. Research indicates that estrogen protects female subjects from aortic aneurysms by reducing oxidative stress and macrophage-associated inflammation [21]. Data preceding the 1990s suggest a protective role of estrogen replacement therapy in reducing aortic stiffness in postmenopausal women [22,23]. Paradoxically, recent clinical evidence derived from a Norwegian cohort has indicated that estrogen replacement therapy rather increased—instead of diminished—the risk of aneurysmal development in women, especially as the time increased after the onset of menopause [19,24]. However, it is worth noting that the effect of female hormone levels was found to be less clinically important when compared with other strong risk factors such as smoking [24].

In accordance with the observed “male disadvantage”, the majority of experimental studies in mice have indicated a strong association of male gonadal sex hormones with AAA development and progression [25,26]. Surprisingly, a piece of recent epidemiological evidence revealed that endogenous testosterone levels are often inversely related, whereas luteinizing hormone levels are positively associated to the risks of aortic aneurysms and other cardiovascular diseases in older men between the ages of 70 to 88 years [27]. Similar protective effects of male hormones were recapitulated in a recent murine study, with the experimental data primarily attributing the observed phenotype to testosterone-mediated anti-inflammatory effects in macrophages, albeit in conflict with the commonly perceived biological functions of testosterone [28]. Conflicting findings in clinical and animal studies suggest that, in male subjects, there may be an optimal physiological threshold of circulating androgens that protects against AAA [19,28].

### 2.3. Anatomical and Biomechanical Factors

There are profound differences in the aortic geometry between males and females which contribute to the many facets of AAA’s sex dimorphism. In the landmark study by B. Rylski et al., aortic geometry changes were characterized throughout life in a cohort free of vascular diseases [29]. Contrasting with earlier studies, the study identified that aortic diameters are greater in males than females only at a young age [30]. Moreover, the dynamic growth of aortas, especially of the aortic arch, is faster in females, which may partially explain the greater rate of AAA dilation and higher rupture risk [29]. The thickening of the arterial walls is believed to be a compensatory mechanism for preventing increased wall stress, particularly as the aortic diameter increases with age. Some clinical findings suggest that the compensatory thickening of the aortic wall is inadequate in males as they age, leading to increased stress on the abdominal aorta [19]. In the same study, male AAA tissues were found to contain more collagen but a much lower elastin content, which is the primary extracellular matrix responsible for the physiological biomechanics in the aorta. This might partially explain why men are more susceptible to initial AAA formation as they age, whereas women remain resistant until menopause [19].

Sex-dependent changes in biomechanical factors may also contribute to the sex dimorphism in AAA. Early studies show that the aortic wall distensibility decreases at an earlier age in males compared to females [31]. Two separate studies by Håkan Åstrand et al. demonstrated that circumferential stress in the abdominal aorta, as well as in other arterial locations such as the common carotid artery, is higher in males than females, as measured by an ultrasound [32,33]. Surprisingly, using cardiovascular magnetic resonance (CMR) to assess PWV and aortic distensibility, R.M. Nethononda et al. report conflicting findings, with females demonstrating a steeper decline in aortic elasticity [34]. J. Tong et al. documented a significantly higher tissue stiffness (modulus) of the luminal thrombi and AAA walls in male versus female patients [35], in agreement with much earlier reports [36]. Indeed, the aortic stiffness in healthy males is greater at all age groups than in females [36]. Aortic stiffness has been an established predisposition factor to various aortopathies including AAA, and the intrinsic sex difference may partially account for the higher prevalence of AAA in males.

Beyond clinical outcomes, the vascular anatomy itself contributes to diagnostic nuances between biological sexes. The use of identical diagnostic criteria for both males and females may lead to the underdiagnosis of a small AAA in females, emphasizing the need to tailor the diagnostic criteria based on gender-specific considerations. This diagnostic challenge has implications for timely intervention and is a critical aspect that necessitates further investigation.

### 2.4. Modifiable Risk Factors: Smoking and Others

Smoking has long been the dominant modifiable risk factor for AAA, with a recent meta-analysis revealing a positive dose–response relationship between the amount of smoking, pack-years smoked, and the risk of AAA [37]. Nevertheless, the vast majority of prior evidence were derived from male subjects, and whether sex-specific responses to smoking are a risk factor remained largely unknown or inconclusive due to the small number of AAA in women. In a recent cross-sectional study comprising 1.5 million female and 0.8 million male subjects, Jennifer L Carter et al. reported a seven-fold increase in the AAA risk in current smoking males over non-smoking, which is on par with the previously reported statistics [18]. Strikingly, in females, current smokers were associated with a risk ratio of 15 when compared with never-smokers, suggesting a 15-fold increase in risk. In women less than 75 years of age, the risk of AAA is ~30 times greater than that of never-smokers. Despite the widely perceived “male disadvantage”, the prevalence of AAA was lower in male never-smokers than female smokers across all age groups. Although not able to be taken into consideration for the 2019 USPSTF recommendation, this milestone study provided strong evidence to consider female smokers as equally, if not more, at risk for AAA. This was later reflected in the 2022 AHA|ACC Joint Guidelines for the Diagnosis and Management of Aortic Disease [17].

Aside from smoking, other strong modifiable risk factors include hypertension, hyperlipidemia, etc. In a recent meta-analysis, Elsa Kobeissi reported a relative risk (RR) of 2.06 in females (hypertensive versus non-hypertensive), which is significantly higher than that in males (RR of 1.46) [38]. Blood lipids such as low-density lipoprotein cholesterol (LDL-C) and triglycerides have also been established to positively correlate with AAA, with similar associations for men and women [18]. Despite early reports suggesting the link between the lipoprotein(a) level and an increased risk of AAA in females, the recent analysis of the ongoing Women’s Health Initiative study reported no such association in this large cohort of postmenopausal women [39]. Lipid-lowering agents, most notably statins, have been subjected to extensive clinical testing; yet, their benefits on controlling AAA remains conflicted [40,41]. Recent evidence supports the postoperative, but not preoperative, use of statins in improving survival after AAA surgical repairs [42]. Although no specific differences have been documented in terms of statins’ impacts on AAA, it is worth noting that the sex bias and underutilization of lipid-lowering drugs have long been documented in female patients with cardiovascular diseases [43]. Other modifiable factors, such as thrombosis and inflammation (e.g., evidenced in the increased C-reactive protein), have been shown to contribute to the prognosis and stratification of AAA, but mostly in male-predominant study populations [44–47]. It is worth noting that diabetes mellitus (DM) status is strongly associated with a lower AAA prevalence in males across numerous studies; however, this protective effect was diminished in females, as shown in an Asian population cohort study [48]. Future efforts are warranted to dissect whether females may respond differently to the modifiable risk factors and related therapies.

### 2.5. Surgical Repairs and Outcomes

As mentioned earlier, female patients exhibit a more complex AAA anatomy and smaller aortic and arterial diameters, resulting in a higher likelihood of undergoing open surgical rather than endovascular repair [35]. To make things worse, the same threshold for surgical repairs of AAA applies to patients of both biological sexes. As reported in a recent meta-analysis, P. Ulug et al. identified that female patients (34%) were less likely to be eligible for endovascular abdominal aortic repair (EVAR) compared to male patients (54%) [6]. Moreover, women are more likely to experience perioperative complications as a result of this less favorable vascular anatomy and standard of operation [7,12]. In fact, the relationship between the aortic diameter and the risk of 30-day mortality varies between male and female patients [49]. Female patients had longer postoperative ICU stays, longer hospital stays, worse 30-day mortality, and more postoperative complications than males [50]. Possibly owing to the shift of the landscape from open repair to EVAR, the in-hospital mortality has witnessed a steady decline over the past two decades [51]. However, female patients are still experiencing higher mortality and complications than males, further fueling the sex disparities in health care options. While a growing body of literature report similar findings of compromised short- and long-term survival in female patients following AAA repairs [52–59], there are conflicting findings that warrant further investigation [55,57]. We have summarized the most relevant studies that shed light onto the sex-specific outcomes post AAA repairs (Table S2).

One likely explanation for the higher mortality rate in women after surgery is the underrecognition of cardiovascular diseases, including AAA, in females [60]. Women may be in a more advanced, undiagnosed medical state than men when they undergo surgery or EVAR. Current practices appear to be less optimal for women, a disparity that is further reflected by the outcomes of AAA repair. AAA in women is less likely to be identified and treated. Growing evidence suggests that non-corrective treatment (“no option” patients) for ruptured AAA is more frequently offered to women than men, regardless of county or age. The decreased mortality related to the treatment of ruptured AAA may suggest a revision of the indication of not screening women for AAA [60]. The improvement in outcomes seen with EVAR compared with open repair has not, unfortunately, eliminated the sex disparity in outcomes [61].

## 3. Preclinical Studies Concerning Sexual Dimorphism in AAA

In contrast to the plethora of existing evidence from clinical studies, experimental efforts toward elucidating the sex dimorphism remain sparse in AAA models.

### 3.1. Sexual Dimorphism in Commonly Used Experimental Models of AAA

To date, multiple animal models have been developed to aid the preclinical research efforts toward a better understanding of AAA. The commonly used AAA murine models each incorporate one or multiple predisposition factors, as similarly observed in the disease process of AAA in patients. The most widely adopted model features the chronic subcutaneous infusion of Angiotensin (Ang) II (induction of hypertension) in a hypercholesterolemic background (high-fat diet in conjunction to loss-of-function in genes such as ApoE and ldlr or gain-of-function in pcsk9) or β-Aminopropionitrile (BAPN) (progressive breakdown of elastic integrity as observed in aged aortic walls) [62,63]. This model is less technically challenging in comparison to the other microsurgery-based ones, but has been widely known to have significant variations and a suboptimal penetrance of the aneurysmal phenotype [64–66]. Moreover, the AngII infusion model generates a saccular aneurysm in the suprarenal aorta, whereas the typical manifestations of clinical AAA should be infrarenal fusiform aortic dilations [66]. Aside from the AngII infusion model, laparotomy procedures can also be conducted to artificially “kick-off” the aortic degeneration and aneurysmal growth, utilizing either a calcium solution (peri-adventitial application of calcium chloride or calcium phosphate to induce aortic calcification, inflammation, and matrix destruction) or porcine pancreatic elastase (intraluminal or peri-adventitial application to induce smooth muscle apoptosis and elastin degradation) [67,68]. To robustly induce an AAA rupture in these aforementioned models, further modification has been introduced with the systemic or topical application of neutralizing antibody targeting Transforming Growth Factor-β (TGF-β), based on prior mechanistic studies supporting the protective role of TGF-β signaling in maintaining aortic homeostasis [69–71]. A newly introduced hypertension-oriented AAA model reports the combination of high salt intake and aberrant activation of the renin–angiotensin–aldosterone system (via putative hypertensive inducers deoxycorticosterone acetate or aldosterone), which offers more clinical relevance in relatively aged mice over the conventional AngII-based models. Other modifiable risk (e.g., smoking/nicotine) and protective factors (e.g., DM) have also been tested in rodents to model the disease process of AAA, in combination with the established inducers [72–75].

Admittedly, none of the experimental models fully recapitulate the human pathophysiology of aortic aneurysmal disease [76]. Nevertheless, they offer valuable insights to aid our interrogation of sexual dimorphism in AAA as well as its cellular and molecular underpinnings. AngII infusion and elastase challenge have both been shown to produce a higher prevalence and greater lesion size of AAA in male versus female mice, although conflicting reports have been reported in the elastase model [62,77,78]. However, none of the animal models recapitulate the “female disadvantage”, as in a faster aneurysmal growth and higher rupture [71]. Unfortunately, to date, no sex differences have been reported in the calcium, high salt, or smoking/nicotine-based AAA models. Non-rodent AAA models are sparse, and even less so in terms of differential phenotypes in male versus female. In a recent report, J Michael Cullen et al. attempted to probe the sex-associated differences in a porcine model of elastase-induced AAA [79]. Surprisingly, while the castration of male pigs led to reduced aneurysmal lesion sizes, no differences in aortic diameters were observed between female and uncastrated male pigs.

### 3.2. Implication of Sex Hormones

Similar to the epidemiological results, data derived from murine models have demonstrated that sex hormones can influence vascular disease development and severity. However, the exact roles of female and male sex hormones remain controversial, with conflicting reports.

#### Female sex hormones:

Like the observations from the Nord-Trøndelag Health Study (HUNT) study of Norwegian women, estrogen has been largely attributed as the primary mediator of the “female advantage” in reducing AAA development in experimental models. As first reported in the landmark study by Gorav Ailawadi et al., exogenous estrogen inhibited elastase-induced AAA formation in male rats, and a female-to-male aortic transplant nullified the aortic resistance to aneurysmal dilation [80]. Estrogen receptor alpha (ERα) is the primary mediator of estrogen’s conducive effects, and its expression level has been shown to be inversely correlated with matrix metalloproteinases (MMP) activity and AAA formation [81]. Follow-up studies have then demonstrated the protective effects of other estrogen-boosting approaches, such as selective estrogen receptor modulators (e.g., tamoxifen), a dietary phytoestrogen supplement, etc. [82,83]. Intriguingly, while William Johnston et al. demonstrated that ovariectomy abrogated the female AAA protection in mice, such an outcome was not observed in the report by Tracy Henriques et al. [84,85]. This may be potentially explained by the intrinsic differences between the distinct experimental models in the two studies (elastase versus AngII).

#### Male sex hormones:

While epidemiological data point to a paradoxically protective role for male sex hormones against AAA [27], murine studies mostly point to a disease-driving role for testosterones and the associated downstream signaling [26]. Androgen treatment has been linked with AngII type 1A receptors’ mRNA abundance in abdominal aortas, which may underlie the aggravated development of AAA [86]. Conversely, the castration of male ApoE-deficient mice mitigated the aneurysmal dilation, while the exposure of female ApoE-deficient mice to exogenous testosterone phenocopied the “male disadvantage” upon AngII infusion [86]. Similar phenotypes were also observed in castrated versus uncastrated male pigs following elastase-induced AAA [79]. Interestingly, using a newly created transgenic strain of female mice harboring either XX or XY sex chromosomes, Yasir Alsiraj et al. demonstrated that the Y sex chromosome profoundly exacerbated AAA severity following an AngII challenge [87]. Collectively, evidence derived from these gain- and loss-of-function approaches point to a deleterious role of male sex hormones in AAA pathogenesis. The question of how to reconcile the disparities between clinical and preclinical observations remains a high-priority quest for future endeavors.

### 3.3. Emerging Molecular Cues Behind AAA’s Sex Dimorphism

Going beyond sex hormones, recent studies have unveiled novel genes or pathways that may underlie the documented sex-specific differences in AAA. J K Lee et al. reported that the deficiency of inducible nitric oxide synthase (iNOS), the isoform of the NO-producing enzyme commonly induced during vascular inflammation, substantially promoted the development of AAA in female mice but not in male ones [88]. Conversely, in the study by Peter J. Kuhlencordt et al., the deficiency of the endothelial isoform of NOS, or eNOS, led to an accelerated aortic aneurysm as well as atherosclerosis in male mice over female, although the sex difference was no longer observed after 24 weeks post AngII infusion in the hypercholesterolemic background [89]. Specialized pro-resolving lipid mediators, a superfamily of Omega-3 fatty-acid-derived bioactive lipids, have been widely shown to contribute to vascular homeostasis; and a recent report by Amanda C Filiberto et al. revealed a sex-specific pattern of their abundance and the expression of their associated G-protein coupled receptors [90]. In a different study, Paul D DiMusto et al. documented a sex-specific increase in plasmin activator inhibitor-1 (PAI-1), a serine protease inhibitor highly regulated by female sex hormones, in female versus male mice [91]. Taking advantage of a transgenic male mouse strain harboring XX versus XY sex chromosome, Yasir Alsiraj et al. conducted a microarray assay from murine abdominal aortas, which revealed the enrichment of novel gene sets (e.g., Xist, Npas2, and Bmal1 in XX, versus Cyp2e1 and Kap in XY) that may be implicated in the sex differences behind aortopathy [92]. Most notably, a recent study showed that lysyl oxidase (LOX), responsible for elastin crosslinking and aortic integrity, was subjected to negative regulation by male but not female sex hormones, and its expression level and activity display a profoundly differential enrichment in females versus males in both human and murine AAAs [77]. The administration of a LOX inhibitor, β-Aminopropionitrile (BAPN), abolished the sex dimorphism in the AngII-induced AAA model, further suggesting its critical role behind the aforementioned disparity [77].

## 4. Conclusions

While sex dimorphism in AAA has long been widely acknowledged, our understanding of it remains limited, both clinically and mechanistically. Significant disparities remain not only in the diagnosis and prognosis, but also in the surgical management of AAA in males versus females. Future efforts should be prioritized toward improving precision medicine and developing endovascular devices for female AAA patients, who have historically been underdiagnosed and undertreated. At the molecular and cellular levels, we should acknowledge the lack of understanding behind the sex dimorphism in AAA, and direct more research toward this understudied and underrecognized topic.

## Supplementary Material

Table S1 - region-specific summary

Table S2 - sex-specific mortality v2

The following supporting information can be downloaded at: https://www.mdpi.com/article/10.3390/jvd4010005/s1, Table S1: Region-specific summary, Table S2: Sex-specific mortality
