# Supplementary material for: Sexual Dimorphism in Abdominal Aortic Aneurysm—Insights from Clinical and Experimental Studies
Source: J Vasc Dis. Author manuscript; Available in PMC 2026 Feb 24. (PMC12927653; doi:10.3390/jvd4010005)
Supplement: Table S1 - region-specific summary [file NIHMS2100454-supplement-Table_S1_-_region-specific_summary.pdf]

Region-specific Prevalence of Abdominal Aortic Aneurysm										
**Authors**	**Research Type**	**Publication year**	**Study Period**	**Country**	**Target population**	**Age group**	**Gender**	**Screened Patients**	**Diagnostic Criteria**	**Prevalence**
**North and South America**										
K.L. Summers, et al.	Retrospective Cross-Sectional Study	2021	2001 - 2017	US	General population[1]	45-90 years (mean 67)	Men and women	9,457	Abdominal aortic diameter ≥3.0 cm (ultrasound)	2.8% (men 4.6%， women 1.2%)
W. Tang, et al.	Prospective Cohort Study	2016	1987 - 2013	US	General population	45 - 85 years (mean 54.2)	Men and women	15,703 (men 44.8%, women 55.2%)	Abdominal aortic diameter ≥3.0 cm (no specified methods)	(men 2.52%, women 0.44%)
M. Schermerhorn, et al.	Prospective cross-sectional study	2008	-	USA	Patients referred to medical centers	≥ 65 years	Men and women	2,005	Abdominal aortic diameter ≥3.0 cm (ultrasound)	1.6% (men 2.8%, women 0.2%)
A.B. Newman, et al.	Prospective cohort study	2001	1989 - 1993	USA	General population	All age groups	Men and women	4,734 (men 41.3%, women 58.7%)	Abdominal aortic diameter ≥3.0 cm or infrarenal-to-suprarenal ratio ≥1.2 (ultrasound)	8.8% (men 12.9%, women 5.9%)
C. A. Hinojosa, et al.	Prospective Cross-Sectional Study	2022	2019	Mexico	Patients in medical centers for any reason	≥55 years old in a center ≥ 65 years in all others	Men and women	12,936	Abdominal aortic diameter ≥3.0 cm (CT)	3.08% (men 4.9%, women 1.48%) *calculated based on data provided in referenced study
P. Puech-Leão, et al.	Prospective cross-sectional study	2004	1999	Brazil	General population	≥ 50 years	Men and women	2,756	Abdominal aortic diameter ≥3.0 cm (ultrasound)	2.3% (men 4.6%, women 0.5%)
**Europe**										
B.S. Stacey, et al.	Retrospective Cohort Study	2024	1993 - 2015	UK	General population	1993 – 2003: men aged 60–80 and women aged 65–80. 2003 – 2005: men and women aged 65–80. 2005 -2015：men aged ≥ 65.	Men and women	6,879	Abdominal aortic diameter ≥3.0 cm (ultrasound)	4.0% (men: 6.0%, women: 1.5%)
M.H. Cuong Pham, et al.	Prospective Cohort Study	2024	2010 - 2019	Denmark	General population	≥40 years (mean 62.0)	Men and women	7,442	Abdominal aortic diameter ≥3.0 cm (CTA)	1.6% (men 3.5%, women 0.3%)
I.B. Koncar, et al.	Pilot Screening Project	2024	2023	Serbia	General population	≥50 years (mean 68.8)	Men and women	4,046	Abdominal aortic diameter ≥3.0 cm (ultrasound)	4.8% (men 8.2%, women 1.3%)
C.A. Behrendt, et al.	Prospective Cross-Sectional Study	2023	2016 - 2018	Germany	General population	45 - 74 years (median 61.0)	Men and women	10,000	Abdominal aortic diameter ≥3.0 cm (no specified methods)	0.8%(men 1.26%, women 0.15%）
E. Altobelli, et al.	Prospective Cohort Study	2022	2015 - 2019	Italy	General population	65 - 79 years (mean men 73.6, women 74.3)	Men and women	2,301	Abdominal aortic diameter ≥3.0 cm (ultrasound)	2.7% (men 3.7%, women 0.6%)
A.R. de Boer, et al.	Retrospective Cohort Study	2022	1996 - 2018	Netherlands	Patients with atherosclerotic vascular diseases[7]	40 - 80 years old	Men and women	7,423 (men 73.3%, women 26.7%)	Abdominal aortic diameter ≥3.0 cm (ultrasound)	2.0% (men 2.5%, women 0.7%), with fall in certain age groups through time periods
J. Fite, et al.	Pilot	2021	2017 - 2018	Spain	General	65 years	Men	4,730 (men 50.3%,	Abdominal aortic	Men 1.4%, no

	Screening Study				population		and women	women 44%)	diameter ≥3.0 cm (ultrasound)	AAA in women
A. Duncan, et al.	Prospective Cross-Sectional Study	2021	2016 - 2019	UK	White women with smoking habit or history of coronary disease	65 -74 years (mean 69.6)	Women only	5,169	Abdominal aortic diameter ≥3.0 cm (ultrasound)	Women 0.3%
J. Tkaczyk, et al.	Prospective Cross-Sectional Study	2019	2018	Poland	General population	≥65 years (median 71.4)	Men and women	1,032 (men 44.9%, women 55.1%)	Abdominal aortic diameter ≥3.0 cm (ultrasound or CT)	2.6% (men 4.3%, women 1.2%)
F. Gianfagna, et al.	Prospective Cross-Sectional Study	2018	2013 - 2016	Italy	General population	Men:50 - 75, Women:60 - 75	Men and women	3,755(men 63.7%, women 32.3%)	Abdominal aortic diameter ≥3.0 cm (ultrasound)	2.3% (men 2.3%, women 0.5%)
M. Dahl, et al.	Prospective Observational Study	2018	2011 - 2013	Denmark	General population	Born in 1936, 1941, 1946, 1951	Women only	1,474	Abdominal aortic diameter ≥3.0 cm (ultrasound)	Women 0.7%
T.L. Dereziński, et al.	Prospective Cohort Study	2017	2009 - 2012	Poland	General population	Men ≥60 years Women ≥65 years	Men and women	922	Abdominal aortic diameter ≥3.0 cm (ultrasound)	4.1% (women 0.8%, men aged 60 - 65 years 1.0%, men aged ≥65 years 9.3%)
G. Corrado, et al.	Prospective Cross-Sectional Study	2016	2010 - 2013	Italy	General population	60 - 85 years (mean 62)	Men and women	1,555(men 48.6%, women 51.4%)	Abdominal aortic diameter ≥3.0 cm (ultrasound)	1.4% (men 2.5%, 0.4% women)
M. Ålund, et al.	Retrospective cross-sectional study	2008	1993 - 2005	Sweden	Patients registered in a hospital	All age groups (mean 66.5)	Men and women	5,924 (men 55.0%, women 45.0%)	Abdominal aortic diameter ≥3.0 cm (ultrasound)	3.0% (men 4.2%, women1.5%)
RAP Scott, et al.	Randomized control trial	2002	1998	UK	General population	65 – 80 years	Men and women	9,485	Abdominal aortic diameter ≥3.0 cm (ultrasound)	men 7.6%, women 1.3%
K. Singh, et al.	Prospective cross-sectional study	2001	1994 - 1995	Norway	General population	55 – 74 years	Men and women	6,386 (men 46.4%, women 53.6%)	Abdominal aortic diameter ⩾3.5 cm or infrarenal aortic diameter ⩾5 mm larger than renal aortic diameter or a localized dilatation of aorta (ultrasound)	4.2% (men 8.9%, women 2.2%)
M.H. Seelig, et al.	Prospective cross-sectional study	2000	1993 - 1997	Germany	Patients undergoing transthoracic echocardiography	All ages groups (mean 68.5)	Men and women	13,166 (men 52.8%, women 47.2%)	Abdominal aortic diameter ≥3.0 cm (ultrasound)	0.82% (men 1.3%, women 0.2%)
**Eastern Asia**										
Y. Funamizu, et al.	Prospective Observation Study	2024	2016 - 2017	Japan	Patients scheduled for ultrasound other than AAA	All age groups	Men and women	9,791	Abdominal aortic diameter ≥3.0 cm (ultrasound)	1.3% (men 1.75%, women 0.64%)
H. Kim, et al.	Prospective Cohort Study	2023	2008 - 2019	South Korea	General population	≥50 years (mean 69.0)	Men and women	3,124	Abdominal aortic diameter ≥3.0cm in men or ≥2.8cm in women(ultrasound)	0.7% (men 1.5%, women 0.1%)
W. Li, et al.	Prospective Observational Study	2017	2014 - 2015	China	Patients undergoing coronary angiography for suspected or known coronary artery disease	All age groups (median 64)	Men and women	1,541 (male 70.7%, female 29.3%)	Abdominal aortic diameter ≥3.0 cm (ultrasound or echocardiography)	1.6% (men 2.0%, women 0.7%)
S. Fukuda, et al.	Prospective Cross-Sectional Study	2015	2012 - 2013	Japan	Patients with hypertension	≥60 years (mean 75.0)	Men and women	1,692	Physical & pocket-echo examinations[17]	Mmen 5.2%, women 2.8% *calculated based on data provided in referenced study
W. Guo, et al.	Prospective	2014	2008 -?	China	Patients with or	45 - 80 years	Men	23,810 (men 37.7%,	Abdominal aortic	0.07% (13 cases

	Cohort Study				without hypertension	(mean 62)	and women	women 62.3%)	diameter ≥3.0 cm (ultrasound)	in men, 3 cases in women)
S.H. Oh, et al.	Prospective cross-sectional study	2010	2009	South Korea	Patients undergoing transthoracic echocardiography	All age groups (mean 60.7)	Men and women	4,939 (men 47.9%, women 52.1%)	Abdominal aortic diameter ≥3.0 cm (ultrasound)	0.5% (men 0.97%, women 0.16%)
K. Adachi, et al.	Prospective cross-sectional study	2000	-	Japan	General population	All age groups (mean 68.5)	Men and women	1,591 (men 43.1%, women 56.9%)	Abdominal aortic diameter ≥3.0 cm (ultrasound)	0.3% (4 cases in men, none in women)
**Oceania**										
R. Kee	Retrospective Cohort Study	2024	2018 - 2019	New Zealand	General population	≥50 years (mean 68.0)	Men and women	811	Abdominal aortic diameter ≥3.0 cm (CT)	5.2% (men 7.1%, women 3.1%)
P. Sandiford	Prospective Cross-Sectional Study	2020	2016 - 2018	New Zealand	Māori population only	Men: 54 - 74 years. Women: 65 - 74 years.	Men and women	2,503 (47% male, 53% female)	Abdominal aortic diameter ≥3.0 cm (ultrasound)	2.8% (men 3.8%, women 1.5%)
R. Claridge	Retrospective Cohort Study	2017	2013 - 2014	New Zealand	Patients performed with CTs	All age groups (mean 70.5)	Men and women	3,246 (male 47.3%, female 52.7%)	Abdominal aortic diameter ≥3.0 cm (CT)	5.8% (men 8.7%, women 3.1%)
K.A. McCaul	Randomized Clinical Trial	2016	1996 - 1999[14]	Australia	General population	64 - 83 years	Men only	12,203	Abdominal aortic diameter ≥3.0 cm (ultrasound)	7.2% (men aged 65 - 74 6.6%)
K. Majeed	Retrospective Cross-Sectional Study	2015	2005 - 2011	New Zealand	Patients undergoing transthoracic echocardiography	≥50 years	Men and women	10,403 (male 54.1%, female 45.9%)	Abdominal aortic diameter ≥3.0 cm (transthoracic echocardiography)	3.5% (men 6.8%, women 2.0%)
**Middle East and North Africa**										
F. C. Sevil	Retrospective Cohort Study	2022	2020	Turkey	Patients performed with CTs	All age groups (mean 53.7)	Men and women	5,396	Abdominal aortic diameter ≥3.0 cm (CT)	1.9% (men 3.3%, women 1.6%)
O. Celebi	Retrospective Observational Study	2021	2014 - 2019	Turkey	patients referred for echocardiography	≥18 years (mean 58.0)	Men and women	5,138 (female 43.1%, male 56.9%)	Abdominal aortic diameter ≥3.0 cm (echocardiography)	2.2% (men 3.3%, women 0.75%)
F. Roshanali	Prospective cross-sectional study	2007	2002 - 2004	Iran	Patients undergoing transthoracic echocardiography	All age groups (mean 40.7)	Men and women	1,175 (men 43.4%, 56.6%)	Abdominal aortic diameter ≥4.0 cm (ultrasound)	4.0% (men 4.5%, women 3.6%)
