# Supplementary material for: Sexual Dimorphism in Abdominal Aortic Aneurysm—Insights from Clinical and Experimental Studies
Source: J Vasc Dis. Author manuscript; Available in PMC 2026 Feb 24. (PMC12927653; doi:10.3390/jvd4010005)
Supplement: Table S2 - sex-specific mortality v2 [file NIHMS2100454-supplement-Table_S2_-_sex-specific_mortality_v2.pdf]

Sex-specific Differences in Mortality/Survival Following AAA Repairs

Authors	Publication year	Country/Region	Research Type and Data Source	Repair Type	AAA Presentation	In-hospital/Peri-operative Mortality (%)	Long-term Survival (%)
J. Epple et al.	2024	Germany	Retrospective (health insurance company database)	EVAR	Ruptured AAA	Male 32.2%	Male 40.7% (5-year survival)
						Female 41.5%	Female 29.1% (5-year survival)
S. Saeed et al.	2024	North America	Retrospective (Vascular Quality Initiative/VQI database)	EVAR and Open Repair	Ruptured AAA	Male declining from 38.5% (2003) to 19.6% (2022)	Not reported
						Female declining from 57.1% (2003) to 31.6% (2022)	
B. Li et al.	2022	North America	Retrospective (VQI database)	EVAR and Open Repair	Ruptured AAA	Male 26.6%	Male 49.5% (8-year survival)
						Female 34.4%	Female 36.7% (8-year survival)
V.T. Ho et al.	2021	North America	Retrospective (VQI database)	EVAR	Ruptured AAA	Male 34.5%	Not reported
						Female 45.9%	
C-A. Behrendt et al.	2021	Germany	Retrospective (health insurance company database)	EVAR	Intact AAA	Male 1.7% (in-hospital), 2.4% (30-day)	Similar 5-year survival
						Female 1.6% (in-hospital), 3.0% (30-day)	
R.M.A. Bulder et al.	2020	Sweden	The Swedish National Patient Registry (NPR)	EVAR and Open Repair	Intact AAA	Not reported	Compromised long-term survival in female (1-year, 4-year, and 10-year survival)
N. Ramkumar et al.	2020	North America	Retrospective (VQI database)	EVAR and Open Repair	Intact and ruptured AAA	Not reported	Male 90% and 36% (1-year and 10-year survival)
							Female 87% and 28% (1-year and 10-year survival)
S.E. Deery et al.	2017	United States	American College of Surgeons National Surgical Quality Improvement Program (NSQIP)	EVAR and Open Repair	Intact AAA	Male 1.1% and 4.0% (EVAR and open)	Not reported
						Female 2.9% and 8.2% (EVAR and open)	
R.C. Lo et al.	2013	United States	Vascular Study Group of New England (VSGNE) database	EVAR and Open Repair	Intact and ruptured AAA	Male (30-day mortality):<br>-intact AAA: 0.9% (EVAR) and 1.7% (open)<br>-ruptured AAA: 29% (EVAR) and 34.1% (open)	Male (1-year survival):<br>-intact AAA: 94.5% (EVAR) and 93.9% (open)<br>-ruptured AAA: 63.8% (EVAR) and 59.1% (open)
						Female (30-day mortality):<br>-intact AAA: 1.2% (EVAR) and 3.6% (open)<br>-ruptured AAA: 27.3% (EVAR) and 48.4% (open)	Female (1-year survival):<br>-intact AAA: 95.8% (EVAR) and 92.9% (open)<br>-ruptured AAA: 54.5% (EVAR) and 42.0% (open)
F. Biancari et al.	2011	Finland		Open Repair	Ruptured AAA	Male 60%	90%, 68%, and 45% (1-year, 3-year, and 5-year survival)
						Female 55.5%	
